# Malnutrition predicts severe complications but not survival in laryngeal cancer surgery: a propensity-matched analysis

**DOI:** 10.3389/fnut.2026.1873158

**Published:** 2026-07-23

**Authors:** Guiren Fang, Ting Zhu, Zhenmeng Lin, Mingfang Yan, Zhitao Lin, Shaokun Weng, Cong Bian

**Affiliations:** 1 Department of Head and Neck Surgery, Clinical Oncology School of Fujian Medical University, Fujian Cancer Hospital, NHC Key Laboratory of Cancer Metabolism, Fuzhou, China; 2 Department of CPC, Clinical Oncology School of Fujian Medical University, Fujian Cancer Hospital, NHC Key Laboratory of Cancer Metabolism, Fuzhou, China; 3 Department of Anesthesiology, Clinical Oncology School of Fujian Medical University, Fujian Cancer Hospital, NHC Key Laboratory of Cancer Metabolism, Fuzhou, China

**Keywords:** GLIM criteria, laryngeal cancer, malnutrition, postoperative complications, survival

## Abstract

**Background:**

The impact of preoperative malnutrition on clinical outcomes in patients undergoing laryngeal cancer surgery remains incompletely defined. This study aimed to evaluate the association between malnutrition and postoperative complications, long-term prognosis, and other clinical outcomes, and to compare the predictive performance of the GLIM criteria versus the ESPEN 2015 criteria.

**Methods:**

A retrospective analysis was conducted on prospectively maintained records of 294 laryngeal cancer patients who underwent surgical resection. Malnutrition was diagnosed according to the Global Leadership Initiative on Malnutrition (GLIM) criteria, and the ESPEN 2015 criteria were additionally applied for comparison. The cohort was chronologically divided into a training set (*n* = 191) and a validation set (*n* = 103). Independent risk factors for severe postoperative complications (POCs) were identified using LASSO regression followed by multivariate logistic regression, and a nomogram was constructed and validated. Cox regression analysis was performed to evaluate associations with overall survival. Propensity score matching (PSM) was applied to balance baseline characteristics between the GLIM-malnutrition group and the normal nutrition group, followed by comparative and subgroup analyses.

**Results:**

The prevalence of preoperative malnutrition was 25.9% (76/294) according to GLIM criteria and 13.6% (40/294) according to ESPEN criteria. Malnutrition defined by both criteria was an independent risk factor for severe POCs, and the GLIM criteria demonstrated superior predictive performance. Age >65 years, diabetes, GLIM-malnutrition, and neck dissection were identified as independent risk factors for severe complications. The nomogram constructed based on these predictors exhibited robust predictive performance, with AUC values of 0.803 in the training set and 0.812 in the validation set, along with good calibration and net clinical benefit. In terms of prognosis, the 5-year overall survival rates were 55.0% in the GLIM-malnutrition group and 60.8% in the normal nutrition group, with no significant difference (*p* = 0.077); malnutrition was not an independent prognostic factor. After PSM, the GLIM-malnutrition group showed a significantly higher incidence of severe POCs and a longer postoperative length of stay (PLOS). Subgroup analyses consistently demonstrated more severe POCs and longer PLOS in the GLIM-malnutrition group across all strata, with a greater number of total POCs observed in elderly patients.

**Conclusion:**

Preoperative malnutrition diagnosed by GLIM criteria is highly prevalent in patients undergoing laryngeal cancer surgery and significantly increases the risk of severe complications and prolongs hospital stay. Compared with ESPEN criteria, GLIM criteria show stronger predictive performance for severe postoperative complications. However, preoperative malnutrition does not independently affect long-term overall survival.

## Introduction

Laryngeal cancer remains a significant global health burden, with an estimated 184,615 new cases and 99,840 deaths annually worldwide, accounting for approximately 1.1% of all cancer diagnoses and 1% of cancer-related mortality (1). It accounts for 30–40% of head and neck malignancies, making it the most prevalent neoplasm in otolaryngology (2, 3). Surgery remains the cornerstone of curative treatment for laryngeal cancer, with open surgical approaches – including partial laryngectomy and total laryngectomy – playing a central role in achieving definitive oncological control (4–6).

Malnutrition is prevalent among patients with laryngeal cancer, primarily because tumor-induced mechanical obstruction of the larynx frequently leads to dysphagia and odynophagia, substantially impairing oral intake, while the tumor-driven systemic inflammatory response and metabolic dysregulation promote cancer cachexia, characterized by progressive weight loss, anorexia, and skeletal muscle wasting (7–10). These consequences are profound: malnutrition compromises immune competence, impairs wound healing, diminishes respiratory muscle strength, and predisposes patients to increased risks of postoperative infectious and pulmonary complications, delayed surgical recovery, prolonged hospitalization, and reduced quality of life (11–15).

Despite the well-recognized clinical importance of nutritional status, a universally accepted standard for diagnosing malnutrition in surgical patients has long been lacking, resulting in substantial heterogeneity in reported prevalence and prognostic implications (16). The Global Leadership Initiative on Malnutrition (GLIM) criteria were recently established to address this challenge. This novel tool has been extensively validated across a range of gastrointestinal malignancies – including gastric, esophageal, colorectal, biliary tract, and pancreatic cancers – where it has demonstrated strong predictive value for postoperative outcomes (12, 17–21). However, the applicability and prognostic performance of the GLIM criteria in patients undergoing laryngeal cancer surgery remain unexplored. Therefore, the present study aimed to apply the GLIM criteria to determine the prevalence of preoperative malnutrition and to investigate its impact on severe postoperative complications, long-term survival, and perioperative outcomes, thereby providing an evidence – based reference for perioperative nutritional risk screening and clinical management.

## Methods

### Study design and patient selection

**Figure 1:**
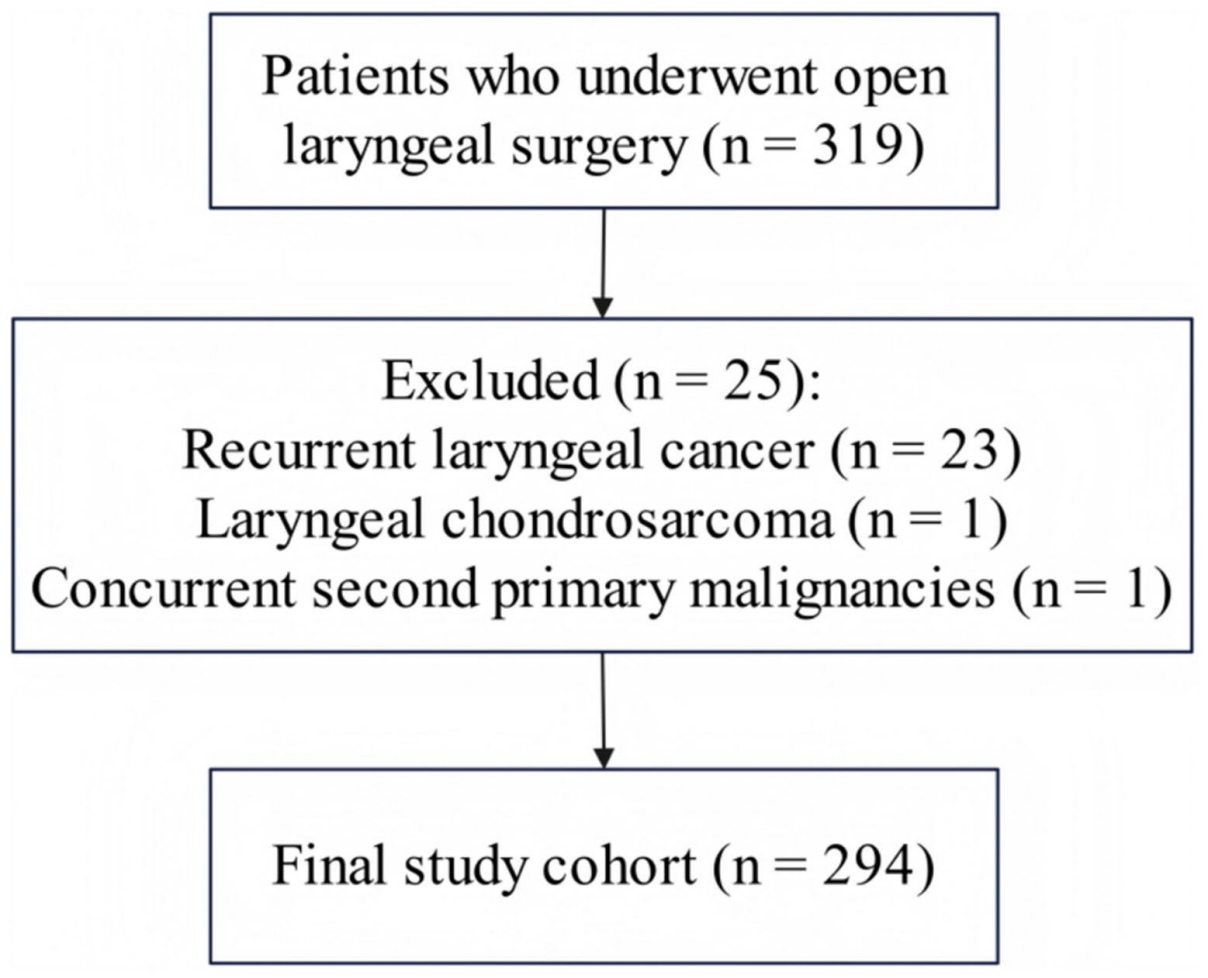
Flowchart of patient selection.

A retrospective analysis was conducted on prospectively maintained clinical records of patients who underwent surgical resection for laryngeal cancer at Fujian Cancer Hospital between November 2014 and December 2023. The inclusion criteria were as follows: (1) histopathologically confirmed squamous cell carcinoma of the larynx; (2) open surgical resection, including partial or total laryngectomy; and (3) complete baseline clinical and laboratory data. The exclusion criteria were as follows: (1) recurrent laryngeal cancer with a history of previous anti-tumor treatment; (2) the presence of distant metastasis at initial diagnosis; and (3) synchronous malignancies at other sites. After applying the above criteria, a total of 294 consecutive patients were ultimately included in the final analysis (Figure 1).

### Variables and definitions

Preoperative nutritional status was evaluated using the two-step GLIM diagnostic framework. First, nutritional risk screening was performed using the Nutritional Risk Screening 2002 (NRS-2002), with a score ≥ 3 indicating nutritional risk. Patients identified as at risk subsequently underwent malnutrition assessment according to GLIM criteria. According to the GLIM criteria, the diagnosis of malnutrition requires at least one phenotypic criterion among the following three: (1) unintentional weight loss, (2) low body mass index (BMI), or (3) reduced muscle mass. Unintentional weight loss was defined as >5% body weight loss within the past 3 months or >10% within the past 6 months. Low BMI was defined as <18.5 kg/m^2^ for patients aged <70 years or <20 kg/m^2^ for those aged ≥70 years. Reduced muscle mass was assessed as a phenotypic criterion. Skeletal muscle mass was measured on preoperative CT images at the C3 level using Slice-O-Matic software, with paravertebral and sternocleidomastoid muscles delineated within −29 to 150 HU (Supplementary Figure 1). The C3 muscle area was converted to the L3 level using the validated Swartz equation, and the skeletal muscle index (SMI) was calculated as estimated L3 muscle area divided by height squared (22, 23). Low muscle mass was defined as SMI < 34.9 cm^2^/m^2^ for females and < 40.8 cm^2^/m^2^ for males (24–26). The etiologic criterion was considered fulfilled in all patients given the inflammatory burden of laryngeal cancer. Patients meeting at least one phenotypic criterion and the etiologic criterion were classified as malnourished.

In addition, we assessed nutritional status according to the 2015 consensus criteria of the European Society for Clinical Nutrition and Metabolism (ESPEN). The diagnostic process consisted of two steps. First, nutritional risk screening was performed using the NRS-2002, and patients with a score ≥ 3 were considered at nutritional risk. Second, for those at risk, malnutrition was diagnosed if either of the following criteria was met: (1) body mass index (BMI) < 18.5 kg/m^2^ regardless of weight loss; or (2) involuntary weight loss (>5% within the past 3 months or >10% within the past 6 months) combined with age-specific low BMI, defined as BMI < 20 kg/m^2^ for patients aged < 70 years or BMI < 22 kg/m^2^ for those aged ≥ 70 years (27).

Postoperative complications (POCs) were graded according to the Clavien-Dindo classification system. Severe POCs were defined as those with a Clavien-Dindo grade ≥ IIIa (28). Given that only one case of subglottic carcinoma was identified in this cohort and its surgical management follows the same principles as that for glottic tumors, glottic and subglottic tumors were combined into a single group. Accordingly, tumors were grouped by anatomical subsite into supraglottic and glottic/subglottic. Age was dichotomized at 65 years, with patients aged >65 years classified as elderly (29). Overall survival (OS) was defined as the time from surgery to death from any cause or last follow-up.

### Surgical procedures

All patients in this cohort underwent open definitive surgical resection for laryngeal cancer, which was categorized into partial laryngectomy and total laryngectomy based on the anatomical extension and clinical stage of the primary tumor. Partial laryngectomy included horizontal supraglottic laryngectomy, vertical partial laryngectomy, or supracricoid partial laryngectomy with cricohyoidopexy (CHP) or cricohyoidoepiglottopexy (CHEP), aiming to preserve laryngeal phonatory and respiratory functions while achieving negative margins. Total laryngectomy was performed for advanced cases where functional preservation was oncologically unfeasible. Concurrent neck dissection was routinely performed for patients with clinically positive lymph nodes or those with a high risk of occult metastasis (such as supraglottic tumors or advanced stage). All surgical interventions were strictly executed or supervised by senior head and neck surgeons following a standardized institutional protocol to ensure consistency in radical resection and laryngeal reconstruction across the study period.

### Statistical analysis

Continuous variables were presented as mean ± standard deviation (SD) or median (interquartile range, IQR) and compared between groups using the independent-samples *t*-test or Mann–Whitney *U* test. Categorical variables were displayed as frequencies and percentages and compared using the chi-square test.

The agreement between GLIM and ESPEN criteria for diagnosing malnutrition was assessed using the Kappa coefficient. Univariate logistic regression was performed to identify factors crudely associated with severe postoperative complications. Subsequently, two separate multivariate logistic regression models were constructed to confirm the independent risk effects of GLIM-defined and ESPEN-defined malnutrition, with consistent adjustment for potential confounders. Random forest analyses were performed separately for GLIM and ESPEN using the same set of covariates; variable importance was evaluated by mean decrease accuracy and mean decrease Gini.

For the development and validation of the predictive model, the whole cohort was divided into a training set and a validation set based on the surgical period. Least absolute shrinkage and selection operator (LASSO) regression was applied to select the most relevant predictors for severe postoperative complications using 10-fold cross-validation. Multivariate logistic regression was then used to identify independent risk factors from the LASSO-selected variables, and a nomogram was constructed. The performance of the nomogram was evaluated by the area under the receiver operating characteristic curve (AUC), calibration curve, Brier score, and decision curve analysis (DCA).

Propensity score matching (PSM) was performed at a 1:2 ratio using the nearest-neighbor method to balance baseline characteristics between the GLIM-malnutrition group and the normal nutrition group. After PSM, between-group comparisons were performed as described above. Survival curves were plotted using the Kaplan–Meier method and compared using the log-rank test. Univariate and multivariate Cox proportional hazards regression analyses were applied to determine independent prognostic factors for overall survival. Subgroup analyses stratified by age, tumor location, and TNM stage were performed to verify the robustness of the results. A two-sided *p*-value < 0.05 was considered statistically significant.

## Results

### Comparison of GLIM and ESPEN malnutrition diagnostic criteria

In the initial NRS-2002 screening, 98 patients (33.3%) were identified as being at nutritional risk (score ≥ 3). The prevalence of preoperative malnutrition was 25.9% (76/294) according to GLIM criteria and 13.6% (40/294) according to ESPEN criteria. Using ESPEN as the reference standard, GLIM achieved a diagnostic accuracy of 81.0%, sensitivity of 75.0%, specificity of 81.9%, and moderate agreement (kappa = 0.413).

Univariate logistic regression indicated that age, diabetes, neck dissection, histological grading, GLIM-malnutrition and ESPEN-malnutrition were all significantly associated with severe postoperative complications (Supplementary Table 1). Two separate multivariate models were established with consistent adjustment for the above confounding factors. After adjustment, both GLIM-malnutrition (OR = 3.083, 95% CI: 1.621–5.863) and ESPEN-malnutrition (OR = 2.723, 95% CI: 1.236–5.998) remained independent risk factors for severe complications (Supplementary Table 2).

Random forest analyses further demonstrated that GLIM-malnutrition yielded higher values in both mean decrease accuracy and mean decrease Gini (Supplementary Figure 2), supporting superior predictive performance of the GLIM criteria for severe postoperative complications.

### Postoperative complications

The overall incidence of POCs was 36.1% (106/294). According to the Clavien-Dindo classification, the severity distribution was as follows: grade I in 28 patients (26.4%), grade II in 21 patients (19.8%), grade III in 52 patients (49.1%), grade IV in 5 patients (4.7%), and grade V (death) in 0 patients (0%). The most common complication was pulmonary infection (45 patients), followed by pharyngeal fistula (38 patients) (Supplementary Table 3).

Furthermore, we performed univariate and multivariate logistic regression analyses to identify independent risk factors for pharyngeal fistula. The results showed that age > 65 years, diabetes, and GLIM-malnutrition were independent risk factors for pharyngeal fistula (Supplementary Table 4).

### Model development and validation

Patients were divided into a training set (November 2014 to December 2020, *n* = 191) and a validation set (January 2021 to December 2023, *n* = 103). The incidence of severe POCs was 39/191 in the training set and 18/103 in the validation set, with no significant difference between the two groups (*p* = 0.543). Baseline characteristics were also comparable between the two sets, as shown in Supplementary Table 5.

**Figure 2:**
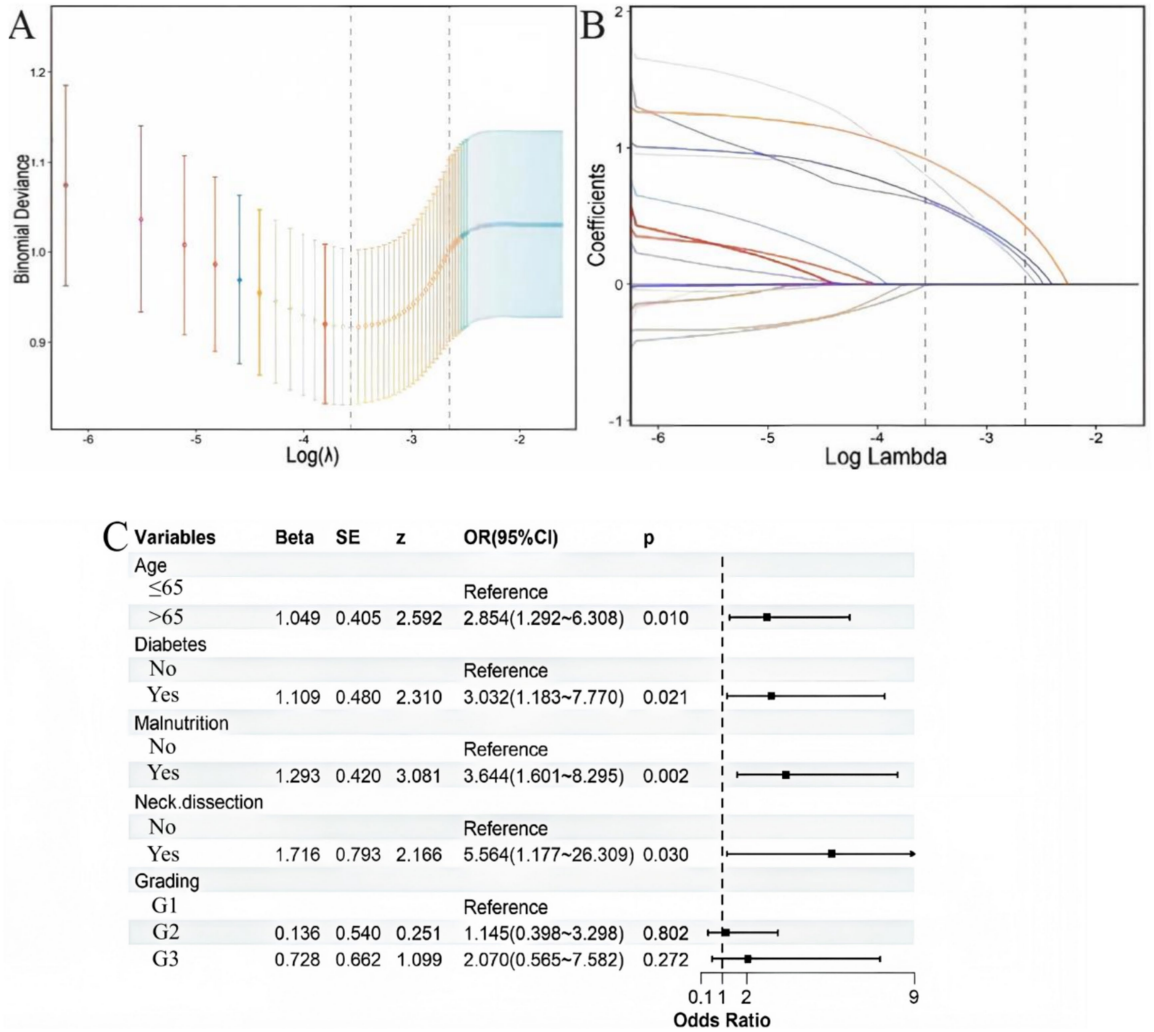
Identification of independent risk factors for severe postoperative complications. **(A)** Binomial deviance plot of the LASSO regression model. **(B)** Coefficient path plots of the LASSO regression model. **(C)** Forest plot of multivariate logistic regression analysis.

**Figure 3:**
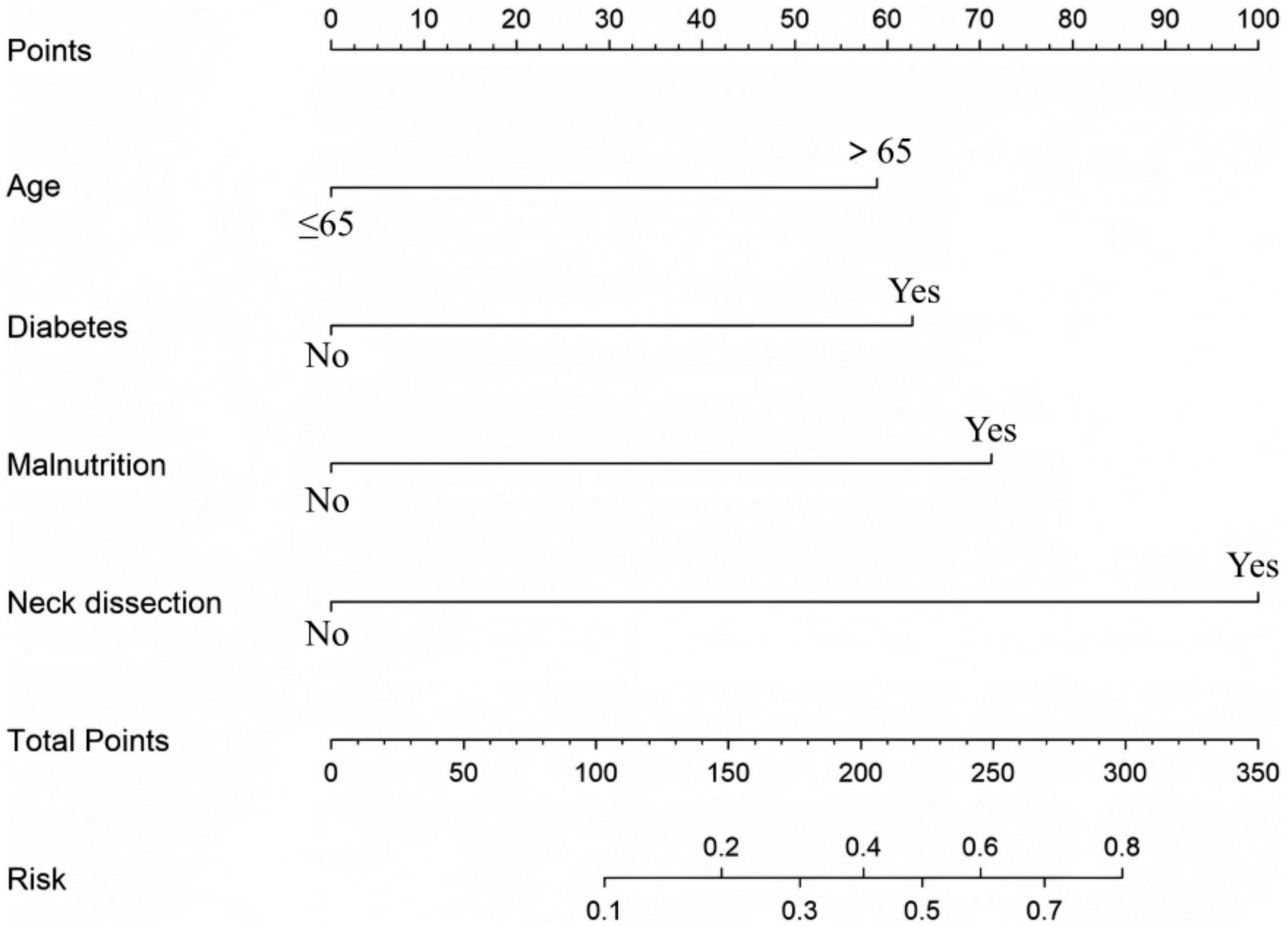
Nomogram for predicting the probability of severe postoperative complications.

**Figure 4:**
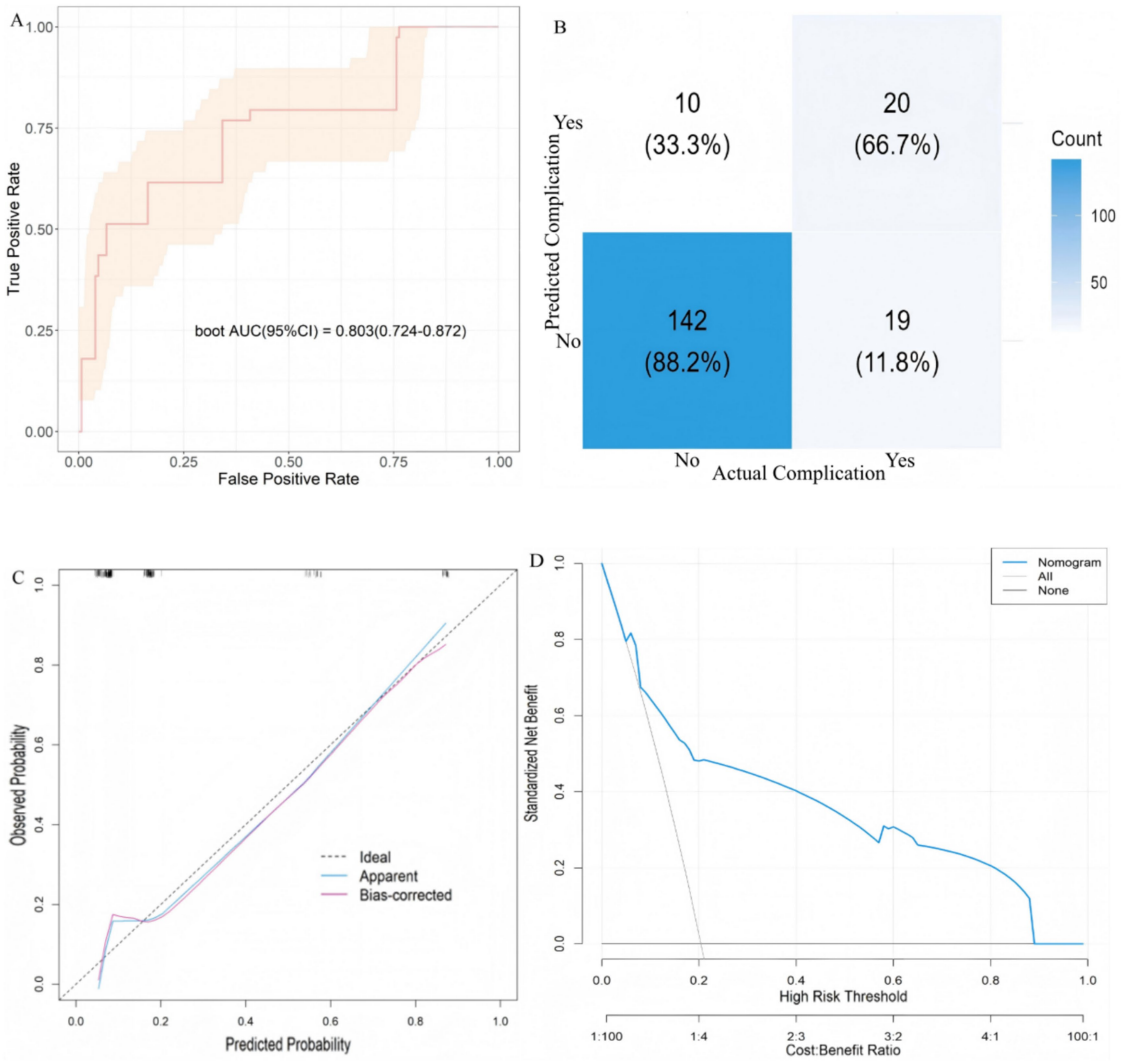
Performance of the nomogram for predicting severe postoperative complications in the training set. **(A)** ROC curve. **(B)** Confusion matrix. **(C)** Calibration curve. **(D)** DCA.

In the training set, LASSO regression identified five potential predictors with non-zero coefficients: age > 65 years, diabetes, GLIM-malnutrition, neck dissection, and histological grading. Subsequently, multivariate logistic regression analysis demonstrated that age > 65 years, diabetes, GLIM-malnutrition, and neck dissection were independent risk factors for severe POCs (Figure 2). A nomogram incorporating these four independent predictors was constructed to estimate the probability of severe POCs (Figure 3). For the training set, the model exhibited good discrimination (AUC = 0.803, 95% CI: 0.724–0.872) and adequate calibration (Brier score = 0.112). Decision curve analysis (DCA) showed net clinical benefit across a threshold probability range of 7–88% (Figure 4 and Supplementary Figure 3). All other performance metrics were within acceptable ranges (Supplementary Table 6).

**Figure 5:**
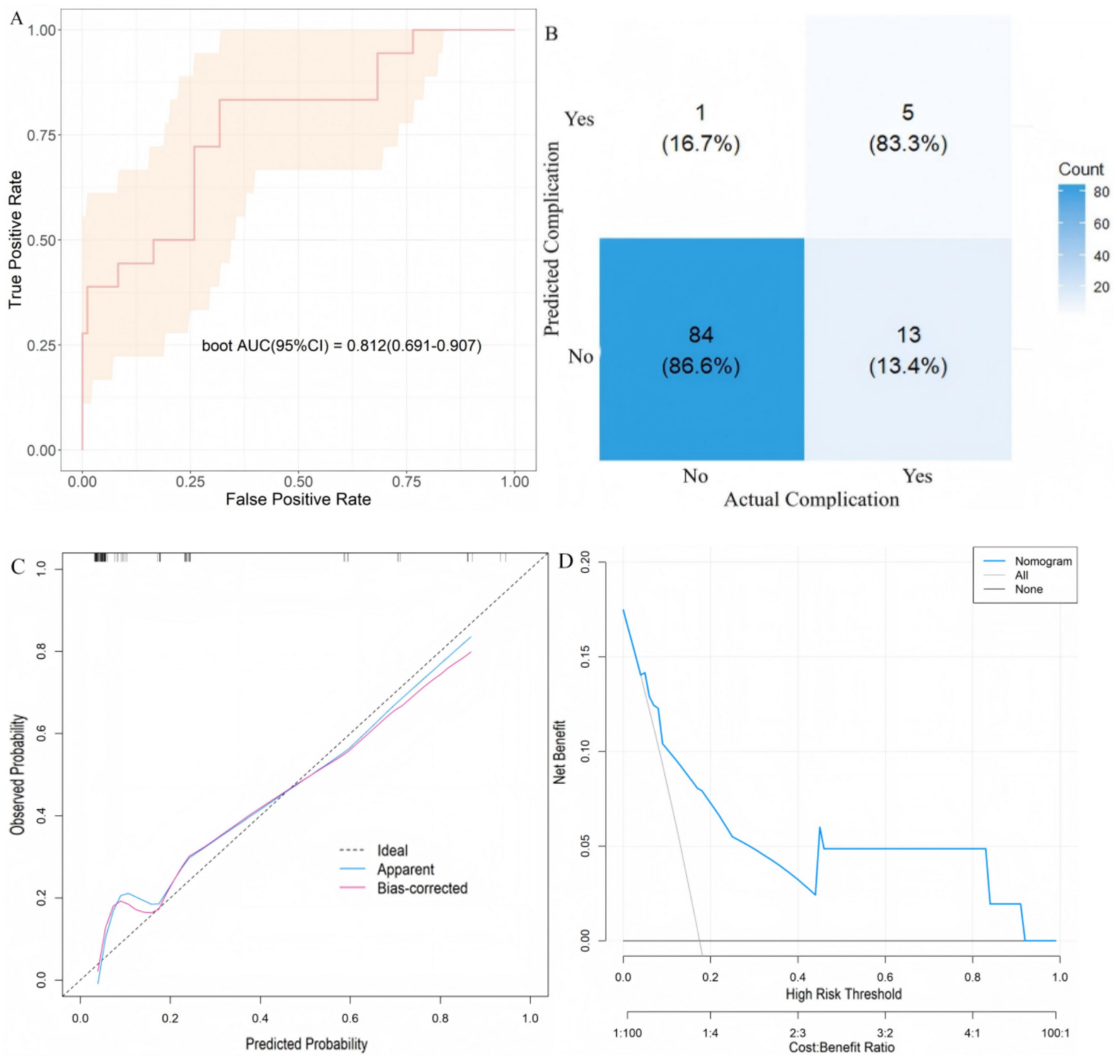
Performance of the nomogram for predicting severe postoperative complications in the validation set. **(A)** ROC curve. **(B)** Confusion matrix. **(C)** Calibration curve. **(D)** DCA.

In the validation set, the model maintained good discriminative power (AUC = 0.812, 95% CI: 0.691–0.907) and calibration (Brier score = 0.087). A net clinical benefit was observed across threshold probabilities ranging from 5 to 91% (Figure 5 and Supplementary Figure 3). Detailed performance metrics are presented in Supplementary Table 6.

### Prognostic analysis

**Figure 6:**
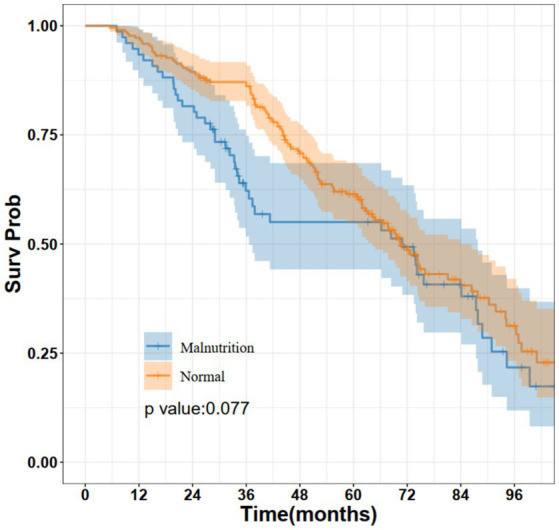
Kaplan–Meier survival curves comparing OS between the GLIM-malnutrition group and the normal nutrition group.

The 5-year OS rates were 55.0% in the GLIM-malnutrition group and 60.8% in the normal nutrition group, with no statistically significant difference between the two groups (*χ*^2^ = 3.126, *p* = 0.077; Figure 6).

Univariate Cox proportional hazards regression analysis identified that neutrophil-to-lymphocyte ratio (NLR), platelet-to-lymphocyte ratio (PLR), histological grading, TNM stage, and perineural invasion were significantly associated with overall survival. These significant variables were subsequently included in a multivariate Cox regression model, which demonstrated that NLR, PLR, TNM stage, and perineural invasion were independent prognostic risk factors for OS (Supplementary Table 7).

### Comparative analysis following propensity score matching

Prior to PSM, GLIM-malnutrition was significantly associated with age, preoperative hemoglobin, preoperative albumin, NLR, PLR, and TNM stage. After PSM, the baseline characteristics between the GLIM-malnutrition group and the normal nutrition group were well balanced (Table 1).

**Table 1:** Baseline characteristics of patients before and after PSM.

Variables	Before PSM			After PSM		
	Malnutrition (*n* = 76)	Normal (*n* = 218)	*p*-value	Malnutrition (*n* = 71)	Normal (*n* = 142)	*P*-value
Age (years)			0.033			0.276
≤65	42 (55.3)	150 (68.8)		39 (54.9)	89 (62.7)	
>65	34 (44.7)	68 (31.2)		32 (45.1)	53 (37.3)	
Sex			0.555			0.312
Male	70 (92.1)	205 (94.0)		65 (91.5)	135 (95.1)	
Female	6 (7.9)	13 (6.0)		6 (8.5)	7 (4.9)	
Smoking			0.149			0.291
Yes	57 (75.0)	144 (66.1)		53 (74.6)	96 (67.6)	
No	19 (25.0)	74 (33.9)		18 (25.4)	46 (32.4)	
Alcohol consumption			0.344			0.383
Yes	40 (52.6)	101 (46.3)		38 (53.5)	67 (47.2)	
No	36 (47.4)	117 (53.7)		33 (46.5)	75 (52.8)	
Hypertension			0.332			0.476
Yes	19 (25.0)	43 (19.7)		17 (23.9)	28 (19.7)	
No	57 (75.0)	175 (80.3)		54 (76.1)	114 (80.3)	
Diabetes			0.426			0.438
Yes	16 (21.1)	37 (17.0)		14 (19.7)	22 (15.5)	
No	60 (78.9)	181 (83.0)		57 (80.3)	120 (84.5)	
Preoperative hemoglobin, g/L, mean (SD)	126.9 ± 20.6	131.8 ± 14.6	0.027	127.8 ± 20.1	131.0 ± 14.1	0.263
Preoperative albumin, g/L, mean (SD)	36.1 ± 4.4	37.5 ± 5.2	0.040	36.1 ± 4.5	36.8 ± 4.8	0.421
PLR, median (IQR)	169.8 [126.9, 219.2]	143.8 [108.9, 195.4]	0.013	170.6 [127.3, 219.3]	158.7 [123.7, 203.8]	0.299
NLR, median (IQR)	2.30 [1.9, 3.4]	2.10 [1.5, 2.9]	0.036	2.40 [1.9, 3.4]	2.20 [1.6, 2.9]	0.101
Surgical period			0.650			0.759
Nov 2014–Dec 2020	51 (67.1)	140 (64.2)		48 (67.6)	93 (65.5)	
Jan 2021–Dec 2023	25 (32.9)	78 (35.8)		23 (32.4)	49 (34.5)	
Surgical approaches			0.575			0.756
Partial laryngectomy	50 (65.8)	151 (69.3)		47 (66.2)	97 (68.3)	
Total laryngectomy	26 (34.2)	67 (30.7)		24 (33.8)	45 (31.7)	
Neck dissection			0.564			0.814
Yes	60 (78.9)	165 (75.7)		55 (77.5)	112 (78.9)	
No	16 (21.1)	53 (24.3)		16 (22.5)	30 (21.1)	
Histological grading			0.255			0.444
G1	14 (18.4)	54 (24.8)		13 (18.3)	34 (23.9)	
G2	48 (63.2)	138 (63.3)		44 (62.0)	88 (62.0)	
G3	14 (18.4)	26 (11.9)		14 (19.7)	20 (14.1)	
Location			0.355			0.689
Supraglottic	31 (40.8)	76 (34.9)		28 (39.4)	52 (36.6)	
Glottic/Subglottic	45 (59.2)	142 (65.1)		43 (60.6)	90 (63.4)	
TNM stage			0.005			0.167
I–II	26 (34.2)	115 (52.8)		24 (33.8)	62 (43.7)	
III–IV	50 (65.8)	103 (47.2)		47 (66.2)	80 (56.3)	
Perineural invasion			0.097			0.228
Yes	14 (18.4)	24 (11.0)		14 (19.7)	19 (13.4)	
No	62 (81.6)	194 (89.0)		57 (80.3)	123 (86.6)	
Vascular invasion			0.070			0.191
Yes	15 (19.7)	25 (11.5)		15 (21.1)	20 (14.1)	
No	61 (80.3)	193 (88.5)		56 (78.9)	122 (85.9)	
Adjuvant treatment			0.158			0.245
Yes	42 (55.3)	100 (45.9)		39 (54.9)	66 (46.5)	
No	34 (44.7)	118 (54.1)		32 (45.1)	76 (53.5)	

PSM, Propensity score matching. PLR, Platelet to lymphocyte Ratio. NLR, Neutrophil to lymphocyte ratio.

**Figure 7:**
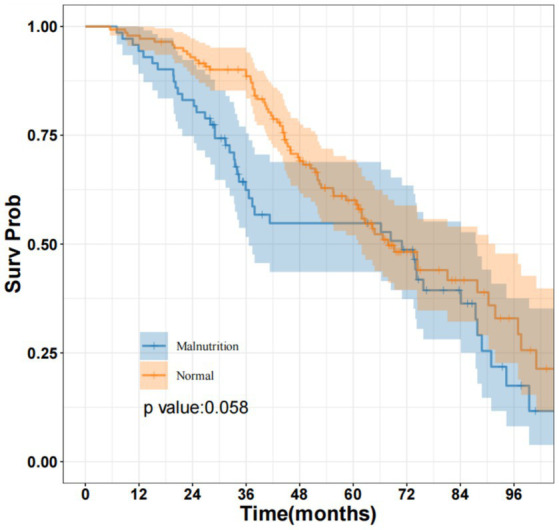
Kaplan–Meier survival curves for OS in the matched cohort.

In the matched cohort, patients with malnutrition had a significantly higher incidence of severe POCs and a longer postoperative length of stay (PLOS) compared with the normal nutrition group. However, no significant differences were observed between the two groups in terms of total POCs, pulmonary infection, pharyngeal fistula, duration of drainage, enteral nutrition duration, positive resection margins, 30-day readmission rate, or 30-day mortality (Table 2). The 5-year OS rates were 54.8% in the GLIM-malnutrition group and 59.1% in the normal nutrition group, with no statistically significant difference (*χ*^2^ = 3.607, *p* = 0.058; Figure 7).

**Table 2:** Comparison of postoperative outcomes between the GLIM-malnutrition and normal groups after PSM.

Outcomes	Malnutrition (*n* = 71)	Normal (*n* = 142)	*P*-value
Total POCs			0.186
Yes	29 (40.8)	45 (31.7)	
No	42 (59.2)	97 (68.3)	
Severe POCs			0.004
Yes	21 (29.6)	19 (13.4)	
No	50 (70.4)	123 (86.6)	
Pulmonary infection			0.272
Yes	13 (18.3)	18 (12.7)	
No	58 (81.7)	124 (87.3)	
Pharyngeal fistula			0.054
Yes	13 (18.3)	13 (9.2)	
No	58 (81.7)	129 (90.8)	
Positive resection margins			0.217
Yes	2 (2.8)	1 (0.7)	
No	69 (97.2)	141 (99.3)	
Duration of drainage, day, median (IQR)	9 (8, 11)	9 (7, 10)	0.159
Enteral nutrition duration, day, median (IQR)	18 (15, 21)	17 (14, 20)	0.074
PLOS, day, mean (SD)	15.4 ± 5.3	12.9 ± 3.7	0.041
30-day readmission rate			0.381
Yes	5 (7.0)	6 (4.2)	
No	66 (93.0)	136 (95.8)	
30-day mortality			-
Yes	0	0	
No	71 (100.0)	142 (100.0)	

POCs, postoperative complications; PLOS, postoperative length of stay.

### Subgroup analysis

Subgroup analyses stratified by age, tumor location, and TNM stage were conducted to further compare clinical outcomes between the GLIM-malnutrition group and the normal nutrition group. Across all subgroups, the GLIM-malnutrition group consistently exhibited a significantly higher incidence of severe POCs and a longer PLOS compared with the normal nutrition group. Regarding total POCs, a statistically significant difference was observed only in the elderly subgroup (age >65 years), with a higher incidence in the GLIM-malnutrition group (Table 3).

**Table 3:** Subgroup analysis of the association between GLIM-malnutrition and postoperative outcomes.

Subgroups	Categories	Patients	Total POCs		Severe POCs		PLOS	
			NO.	*P*-value	NO.	*P*-value	mean (SD)	*P*-value
Age (years)								
≤65	Malnutrition	39	17	0.186	12	0.021	15.0 ± 5.2	0.049
	Normal	89	28		12		13.2 ± 4.2	
>65	Malnutrition	32	16	0.016	10	0.023	16.0 ± 5.6	<0.001
	Normal	53	13		6		12.3 ± 2.4	
Location								
Supraglottic	Malnutrition	28	11	0.342	6	0.034	16.0 ± 6.1	0.003
	Normal	52	15		3		12.6 ± 3.6	
Glottic/Subglottic	Malnutrition	43	20	0.084	17	0.002	15.1 ± 4.9	0.009
	Normal	90	28		14		13.0 ± 3.7	
TNM stage								
I–II	Malnutrition	24	11	0.546	10	0.021	15.5 ± 5.0	0.033
	Normal	62	24		11		13.4 ± 3.6	
III–IV	Malnutrition	47	18	0.155	11	0.041	15.5 ± 5.6	<0.001
	Normal	80	21		8		12.4 ± 3.7	

POCs, postoperative complications. PLOS, postoperative length of stay.

## Discussion

The prevalence of preoperative malnutrition diagnosed by the GLIM criteria was 25.9% (76/294), compared to 13.6% (40/294) by the ESPEN criteria, highlighting a substantial nutritional burden in this population. Notably, GLIM identified a higher malnutrition rate than ESPEN. This discrepancy may be partly explained by the fact that ESPEN criteria are less sensitive in detecting malnourished patients with a normal or high baseline body mass index and muscle mass. Importantly, GLIM-malnourished patients exhibited significantly lower preoperative hemoglobin and serum albumin levels than their well-nourished counterparts. Hemoglobin and serum albumin are well-established biochemical markers reflecting nutritional reserves and systemic nutritional status: hemoglobin synthesis depends directly on adequate protein and iron intake, whereas hepatic albumin production requires sufficient amino acid substrates (30, 31). The significant differences in these two nutritional markers between groups provide objective validation of the reliability and validity of the GLIM criteria for diagnosing malnutrition in patients undergoing open laryngeal cancer surgery.

GLIM-malnutrition in our cohort was significantly associated with older age and advanced TNM stage (III–IV). This observation is biologically plausible: elderly patients typically have diminished physiological reserve and compensatory capacity, rendering them more vulnerable to the metabolic stress imposed by malignancy (32, 33), while patients with advanced-stage disease often experience more severe tumor-related symptoms – such as dysphagia, odynophagia, and anorexia – together with greater tumor – driven metabolic demands, which accelerate nutritional depletion and lead to a higher prevalence of malnutrition (34). Our data further showed that NLR and PLR were significantly higher in malnourished patients than in those with normal nutrition. NLR and PLR are well-established composite indicators reflecting systemic inflammation and immune competence in patients with cancer. Malnutrition can trigger a systemic inflammatory response and immune dysregulation in patients with laryngeal cancer (35, 36). Consistent with evidence from multiple solid tumor types (37–41), our multivariate Cox regression analysis further confirmed that preoperative NLR and PLR were independent prognostic factors for OS in patients with laryngeal cancer, underscoring the critical role of systemic inflammation in determining long-term oncologic outcomes.

In the present study, we focused our primary analysis on severe POCs rather than total POCs, because severe complications – requiring interventional management or being life-threatening-directly jeopardize patient safety, prolong hospitalization, and increase healthcare costs (42, 43). Using severe POCs as the reference outcome, we compared the predictive performance of the GLIM criteria with that of the ESPEN 2015 criteria. Although both malnutrition definitions were independent risk factors for severe complications in multivariate logistic regression, GLIM-malnutrition yielded a higher odds ratio (OR = 3.083) than ESPEN-malnutrition (OR = 2.723). Moreover, random forest variable importance analysis demonstrated that GLIM contributed more to the prediction of severe POCs than ESPEN, as reflected by higher mean decrease accuracy and mean decrease Gini values. These findings suggest that, when benchmarked against a clinically meaningful endpoint – severe postoperative complications – the GLIM criteria provide superior risk stratification for patients undergoing laryngeal cancer surgery compared to the ESPEN criteria.

Our findings consistently identified malnutrition as an independent risk factor for severe POCs in the overall cohort, a result corroborated by both propensity score matching and subgroup analyses, in which malnourished patients experienced significantly more severe POCs and a longer postoperative length of stay. Several mechanisms may underlie this association. Preoperative malnutrition leads to depletion of protein and energy reserves, which directly suppresses cellular and humoral immunity and reduces the host’s defense against postoperative infections. Meanwhile, inadequate nutrient supply impairs fibroblast proliferation and tissue repair, delaying wound healing and increasing the risk of surgery-related complications. Furthermore, laryngeal cancer itself induces a persistent systemic inflammatory state, and malnutrition further amplifies this inflammation while compromising respiratory muscle function. Collectively, these factors predispose patients to severe adverse events (13, 44–46). Notably, a significant difference in total POCs was observed only in elderly patients, suggesting that older individuals may be particularly sensitive to the totality of malnutrition-related complications due to diminished physiological reserve.

Beyond malnutrition, our multivariate analysis identified age >65 years, diabetes, and neck dissection as additional independent risk factors for severe POCs. Advanced age is associated with diminished physiological reserve, impaired immune function, and delayed tissue repair, collectively increasing susceptibility to surgical complications (47, 48). Diabetes contributes to microvascular dysfunction, compromised wound healing, and heightened infection risk (49, 50). Neck dissection involves extensive tissue manipulation, which increases surgical trauma and the risk of lymphatic leakage and infectious complications (51).

A nomogram based on these independent risk factors was constructed to estimate the probability of severe POCs. The model demonstrated satisfactory discrimination, calibration, and clinical utility in both the training and validation sets. By integrating readily available preoperative variables, this nomogram provides a practical bedside tool for individualized risk stratification, enabling clinicians to identify high-risk patients and implement targeted preoperative optimization strategies before laryngeal cancer surgery.

In the present study, GLIM-malnutrition was not an independent prognostic factor for long-term survival, and no significant difference in 5-year OS was observed between the malnutrition and normal nutrition groups in either the overall or the propensity score-matched cohort. Although preoperative malnutrition acts as an amplifier of acute stress, triggering immediate and severe postoperative complications, it does not alter the underlying genomic and inflammatory background of the disease. Once patients successfully navigate the acute perioperative risk period, their long-term survival is primarily governed by these dominant oncological drivers – tumor stage, perineural invasion, and systemic inflammation – rather than by baseline nutritional status alone. The prognostic value of GLIM-malnutrition remains controversial across cancer types. Studies in gastric and colorectal cancers have demonstrated a significant association between GLIM-malnutrition and worse long-term survival, whereas consistent with our findings, evidence in esophageal cancer suggests no independent relationship (52–56). This discrepancy may reflect differences in the dominant prognostic drivers specific to each tumor type: in laryngeal cancer, survival appears to be governed more heavily by tumor biology, disease stage, and systemic inflammatory status rather than by preoperative nutritional status alone.

Several limitations of this study should be acknowledged. First, this was a single-center study with a relatively modest sample size, which may affect statistical power and the generalizability of the findings. Nevertheless, given the relatively low incidence of laryngeal cancer and the fact that many patients receive non-surgical treatments, a cohort of 294 patients collected at a single institution has important clinical significance. Second, although clinical data were prospectively collected and maintained, the present analysis was conducted retrospectively and may be subject to selection and information biases. Third, the study enrolled patients over a lengthy time span. However, during this period, there were no significant changes in surgical techniques, instruments, or perioperative management protocols for laryngeal cancer at our institution, thereby minimizing major temporal heterogeneity. Fourth, transoral laser microsurgery and transoral robotic surgery, which have become increasingly adopted as minimally invasive treatment modalities for early-stage laryngeal cancer (57, 58), have not yet been implemented at our institution, limiting the applicability of our findings to populations treated with these approaches. Fifth, the nomogram for predicting severe POCs was developed and internally validated using a single-center dataset without external validation. Although the model demonstrated favorable discrimination and calibration, its predictive performance and clinical applicability in other surgical populations remain to be established. Sixth, only complete OS data were available; reliable disease-free survival (DFS) data could not be obtained due to the long follow-up period and patients’ inaccurate recall of recurrence time, which limited the evaluation of malnutrition’s impact on tumor recurrence. Additionally, the number of positive resection margin events was very small in this cohort, which limited the statistical power to explore the potential association between preoperative malnutrition and surgical margin status.

## Conclusion

Preoperative malnutrition defined by the GLIM criteria is highly prevalent among patients undergoing open laryngeal cancer surgery, significantly increasing the risk of severe postoperative complications and prolonging hospital stay, but does not independently affect long-term overall survival. The nomogram constructed in this study demonstrated favorable predictive performance and clinical utility for severe postoperative complications, providing a valuable tool for identifying high-risk patients and guiding targeted preoperative optimization strategies.

## Data Availability

The original contributions presented in the study are included in the article/Supplementary material, further inquiries can be directed to the corresponding author.
