# Supplementary material for: Malnutrition predicts severe complications but not survival in laryngeal cancer surgery: a propensity-matched analysis
Source: Front Nutr. 2026 Jul 23;13:1873158. doi: 10.3389/fnut.2026.1873158 (PMC13445962; doi:10.3389/fnut.2026.1873158)
Supplement: Supplementary file 1 [file Data_sheet_1.docx]

SUPPLEMENTARY FIGURE 1

Measurement of skeletal muscle area at the C3 level using Slice-o-Matic software.

**SUPPLEMENTARY TABLE 1 Univariate analysis of factors associated with severe postoperative complications.**

| Variables | OR (95%CI) | P value |
| --- | --- | --- |
| Age |  | 0.001 |
| ≤65 | Reference |  |
| >65 | 2.788 (1.544-5.032) |  |
| Sex |  | 0.433 |
| Male | Reference |  |
| Female | 1.532 (0.528-4.442) |  |
| Smoking |  | 0.113 |
| Yes | Reference |  |
| No | 0.580 (0.295-1.139) |  |
| Alcohol consumption |  | 0.432 |
| Yes | Reference |  |
| No | 0.793 (0.444-1.415) |  |
| Hypertension |  | 0.153 |
| Yes | Reference |  |
| No | 0.617 (0.319-1.196) |  |
| Diabetes |  | 0.011 |
| No | Reference |  |
| Yes | 2.373 (1.215-4.634) |  |
| GLIM-malnutrition |  | <0.001 |
| No | Reference |  |
| Yes | 3.453 (1.881-6.340) |  |
| ESPEN-malnutrition |  | 0.027 |
| No | Reference |  |
| Yes | 2.298 (1.100-4.803) |  |
| Preoperative hemoglobin | 0.990 (0.973-1.008) | 0.277 |
| Preoperative albumin | 0.956 (0.900-1.016) | 0.146 |
| NLR | 1.003 (0.999-1.006) | 0.139 |
| PLR | 1.140 (0.972-1.336) | 0.108 |
| Surgical approaches |  | 0.347 |
| Partial laryngectomy | Reference |  |
| Total laryngectomy | 1.337 (0.730-2.449) |  |
| Neck dissection |  | 0.014 |
| No | Reference |  |
| Yes | 3.078 (1.259-7.522) |  |
| Histological grading |  |  |
| G1 | Reference |  |
| G2 | 1.297 (0.602-2.794) | 0.506 |
| G3 | 2.793 (1.088-7.166) | 0.033 |
| Location |  | 0.700 |
| Supraglottic | Reference |  |
| Glottic / Subglottic | 0.890 (0.490-1.614) |  |
| TNM stage |  | 0.117 |
| I-II | Reference |  |
| III-IV | 1.604 (0.889-2.897) |  |
| Perineural invasion |  | 0.114 |
| Yes | Reference |  |
| No | 0.538 (0.249-1.162) |  |
| Vascular invasion |  | 0.072 |
| Yes | Reference |  |
| No | 0.502 (0.238-1.063) |  |

NLR: Neutrophil to lymphocyte ratio. PLR: Platelet to Lymphocyte Ratio.

**SUPPLEMENTARY TABLE** 2 Multivariable logistic regression analysis of risk factors for severe postoperative complications.

| Risk Factors | Model 1(GLIM) | |  | Model 2 (ESPEN ) | |
| --- | --- | --- | --- | --- | --- |
|  | OR (95%CI) | p |  | OR (95%CI) | p |
| Age | 2.573(1.373-4.820) | 0.003 |  | 2.913(1.567-5.416) | <0.001 |
| Diabetes | 2.246(1.081-4.670) | 0.030 |  | 2.570(1.261-5.239) | 0.009 |
| Malnutrition | 3.083(1.621-5.863) | 0.001 |  | 2.723(1.236-5.998) | 0.013 |
| Neck dissection | 2.568(1.005-6.557) | 0.049 |  | 2.726(1.063-6.986) | 0.037 |
| Histological grading | 1.357(0.729-5.705) | 0.175 |  | 2.063(0.750-5.676) | 0.161 |

Model 1 was adjusted for age, diabetes, neck dissection, and histological grading, with GLIM-malnutrition as the nutritional variable. Model 2 was adjusted for the same set of covariates, with ESPEN-malnutrition as the nutritional variable.

Supplementary Figure 2. Variable importance of random forest models for predicting severe postoperative complications. (A) Random forest model incorporating GLIM-malnutrition.(B) Random forest model incorporating ESPEN-malnutrition.

**Supplementary Table 3 Types and frequencies of postoperative complications**

| Complication | Number of patients | Rate (%) |
| --- | --- | --- |
| Pulmonary infection | 45 | 15.3 |
| Pharyngeal fistula | 38 | 12.9 |
| Wound infection | 38 | 12.9 |
| Dysphagia | 36 | 12.2 |
| Laryngeal granuloma | 7 | 2.4 |
| Acute respiratory failure | 3 | 1.0 |

**SUPPLEMENTARY TABLE 4 Univariate and multivariate logistic regression analyses of risk factors for pharyngeal fistula.**

| Variables | Univariate analysis | | Multivariate analysis | |
| --- | --- | --- | --- | --- |
|  | OR (95%CI) | P value | OR (95%CI) | P value |
| Age |  | 0.001 |  | 0.001 |
| ≤65 | Reference |  | Reference |  |
| >65 | 3.435(1.702-6.935) |  | 3.463(1.679-7.139) |  |
| Sex |  | 0.282 |  |  |
| Male | Reference |  |  |  |
| Female | 1.890(0.593-6.029) |  |  |  |
| Smoking |  | 0.262 |  |  |
| Yes | Reference |  |  |  |
| No | 0.635(0.288-1.403) |  |  |  |
| Alcohol consumption |  | 0.336 |  |  |
| Yes | Reference |  |  |  |
| No | 0.714(0.360-1.417) |  |  |  |
| Hypertension |  | 0.093 |  |  |
| Yes | Reference |  |  |  |
| No | 0.526(0.248-1.114) |  |  |  |
| Diabetes |  | 0.007 |  | 0.006 |
| Yes | Reference |  | Reference |  |
| No | 0.356(0.168-0.754) |  | 0.331(0.150-0.731) |  |
| Malnutrition |  | 0.016 |  | 0.030 |
| Yes | Reference |  | Reference |  |
| No | 0.421(0.208-0.853) |  | 0.441(0.210-0.924) |  |
| Preoperative hemoglobin | 0.988(0.968-1.009) | 0.257 |  |  |
| Preoperative albumin | 0.935(0.869-1.007) | 0.075 |  |  |
| NLR | 1.003(0.999-1.007) | 0.127 |  |  |
| PLR | 1.138(0.951-1.362) | 0.159 |  |  |
| Surgical approaches |  | 0.267 |  |  |
| Partial laryngectomy | Reference |  |  |  |
| Total laryngectomy | 1.488(0.737-3.006) |  |  |  |
| Neck dissection |  | 0.116 |  |  |
| Yes | Reference |  |  |  |
| No | 0.455(0.170-1.214) |  |  |  |
| Histological grading |  |  |  |  |
| G1 | Reference |  |  |  |
| G2 | 0.790(0.341-1.831) | 0.582 |  |  |
| G3 | 1.903(0.686-5.284) | 0.217 |  |  |
| Location |  | 0.673 |  |  |
| Supraglottic | Reference |  |  |  |
| Glottic / Subglottic | 0.860(0.428-1.730) |  |  |  |
| TNM stage |  | 0.264 |  |  |
| I-II | Reference |  |  |  |
| III-IV | 1.486(0.742-2.978) |  |  |  |
| Perineural invasion |  | 0.283 |  |  |
| Yes | Reference |  |  |  |
| No | 0.610(0.248-1.504) |  |  |  |
| Vascular invasion |  | 0.674 |  |  |
| Yes | Reference |  |  |  |
| No | 0.817(0.318-2.099) |  |  |  |

PLR: Platelet to Lymphocyte Ratio. NLR: Neutrophil to lymphocyte ratio.

**SUPPLEMENTARY TABLE 5 Comparison of baseline characteristics between the training set and validation set.**

| Variables | Training set  (n=191) | Validation set (n=103) | P value |
| --- | --- | --- | --- |
| Age (years) |  |  | 0.745 |
| ≤65 | 126 (66.0) | 66 (64.1) |  |
| >65 | 65 (34.0) | 37 (35.9) |  |
| Sex |  |  | 0.410 |
| Male | 177 (92.7) | 98 (95.1) |  |
| Female | 14 ( 7.3) | 5 ( 4.9) |  |
| Smoking |  |  | 0.154 |
| Yes | 136 (71.2) | 65 (63.1) |  |
| No | 55 (28.8) | 38 (36.9) |  |
| Alcohol consumption |  |  | 0.260 |
| Yes | 87 (45.5) | 54 (52.4) |  |
| No | 104 (54.5) | 49 (47.6) |  |
| Hypertension |  |  | 0.415 |
| Yes | 43 (22.5) | 19 (18.4) |  |
| No | 148 (77.5) | 84 (81.6) |  |
| Diabetes |  |  | 0.084 |
| Yes | 29 (15.2) | 24 (23.3) |  |
| No | 162 (84.8) | 79 (76.7) |  |
| Preoperative hemoglobin, g/L, mean (SD) | 129.9 ± 15.8 | 131.6 ± 17.8 | 0.408 |
| Preoperative albumin, g/L, mean (SD) | 37.3 ± 5.1 | 36.8 ± 4.9 | 0.463 |
| NLR, median (IQR) | 2.20 [1.50, 3.15] | 2.20 [1.60, 2.95] | 0.860 |
| PLR, median (IQR) | 148.5 [116.8, 200.9] | 148.7 [105.6, 205.4] | 0.435 |
| Surgical approaches |  |  | 0.154 |
| Partial laryngectomy | 136 (71.2) | 65 (63.1) |  |
| Total laryngectomy | 55 (28.8) | 38 (36.9) |  |
| Neck dissection |  |  | 0.360 |
| Yes | 143 (74.9) | 82 (79.6) |  |
| No | 48 (25.1) | 21 (20.4) |  |
| Histological grading |  |  | 0.478 |
| G1 | 40 (20.9) | 28 (27.2) |  |
| G2 | 124 (64.9) | 62 (60.2) |  |
| G3 | 27 (14.1) | 13 (12.6) |  |
| Location |  |  | 0.057 |
| Supraglottic | 77 (40.3) | 30 (29.1) |  |
| Glottic / Subglottic | 114 (59.7) | 73 (70.9) |  |
| TNM stage |  |  | 0.732 |
| I-II | 93 (48.7) | 48 (46.6) |  |
| III-IV | 98 (51.3) | 55 (53.4) |  |
| Perineural invasion |  |  | 0.053 |
| Yes | 30 (15.7) | 8 ( 7.8) |  |
| No | 161 (84.3) | 95 (92.2) |  |
| Vascular invasion |  |  | 0.155 |
| Yes | 22 (11.5) | 18 (17.5) |  |
| No | 169 (88.5) | 85 (82.5) |  |

**SUPPLEMENTARY TABLE 6 Performance metrics of the nomogram for predicting severe POCs in the training and validation sets.**

| Metric | Training set (n=191) | Validation set (n=103) |
| --- | --- | --- |
| AUC | 0.803(0.724-0.882) | 0.812(0.703-0.921) |
| Specificity | 0.836 | 0.682 |
| Sensitivity | 0.615 | 0.833 |
| Accuracy | 0.791 | 0.709 |
| NPV | 0.894 | 0.951 |
| Precision | 0.490 | 0.357 |
| F1 | 0.545 | 0.500 |

NPV: Negative predictive value.

SUPPLEMENTARY FIGURE 3

Supplementary evaluation of the nomogram. (A) Radar chart summarizing performance metrics in the training set. (B) Clinical impact curve in the training set. (C) Radar chart summarizing performance metrics in the validation set. (D) Clinical impact curve in the validation set.

**SUPPLEMENTARY TABLE 7 Univariate and multivariate Cox regression analysis of prognostic factors for overall survival.**

| Variables | Univariate analysis | | Multivariate analysis | |
| --- | --- | --- | --- | --- |
|  | HR (95%CI) | P value | HR (95%CI) | P value |
| Age |  | 0.144 |  |  |
| ≤65 | Reference |  |  |  |
| >65 | 1.281 (0.919-1.786) |  |  |  |
| Sex |  | 0.641 |  |  |
| Male | Reference |  |  |  |
| Female | 1.146 (0.647-2.029) |  |  |  |
| Smoking |  | 0.098 |  |  |
| Yes | Reference |  |  |  |
| No | 0.749 (0.533-1.054) |  |  |  |
| Alcohol consumption |  | 0.464 |  |  |
| Yes | Reference |  |  |  |
| No | 0.891 (0.653-1.215) |  |  |  |
| Hypertension |  | 0.635 |  |  |
| Yes | Reference |  |  |  |
| No | 0.910 (0.617-1.342) |  |  |  |
| Diabetes |  | 0.086 |  |  |
| Yes | Reference |  |  |  |
| No | 0.714 (0.487-1.049) |  |  |  |
| Malnutrition |  | 0.077 |  |  |
| Yes | Reference |  |  |  |
| No | 0.735 (0.521-1.036) |  |  |  |
| Preoperative hemoglobin | 0.993 (0.984-1.003) | 0.187 |  |  |
| Preoperative albumin | 0.973 (0.944-1.003) | 0.074 |  |  |
| NLR | 1.003 (1.001-1.005) | 0.001 | 1.003 (1.001-1.005) | 0.009 |
| PLR | 1.186 (1.100-1.279) | <0.001 | 1.111 (1.016-1.215) | 0.020 |
| Surgical approaches |  | 0.581 |  |  |
| Partial laryngectomy | Reference |  |  |  |
| Total laryngectomy | 1.098 (0.787-1.532) |  |  |  |
| Neck dissection |  | 0.086 |  |  |
| Yes | Reference |  |  |  |
| No | 0.715 (0.488-1.048) |  |  |  |
| Histological grading |  |  |  |  |
| G1 | Reference |  |  |  |
| G2 | 1.105 (0.747-1.633) | 0.618 | 1.054 (0.711-1.561) | 0.795 |
| G3 | 1.818 (1.100-3.005) | 0.020 | 1.268 (0.753-2.135) | 0.373 |
| Location |  | 0.401 |  |  |
| Supraglottic | Reference |  |  |  |
| Glottic / Subglottic | 1.152 (0.828-1.602) |  |  |  |
| TNM stage |  | <0.001 |  | <0.001 |
| I-II | Reference |  | Reference |  |
| III-IV | 2.835 (2.022-3.974) |  | 2.395 (1.650-3.477) |  |
| Perineural invasion |  | <0.001 |  | 0.034 |
| Yes | Reference |  | Reference |  |
| No | 0.414 (0.268-0.638) |  | 0.613 (0.390-0.964) |  |
| Vascular invasion |  | 0.055 |  |  |
| Yes | Reference |  |  |  |
| No | 0.651 (0.420-1.009) |  |  |  |
| Severe POCs |  | 0.069 |  |  |
| Yes | Reference |  |  |  |
| No | 0.710 (0.490-1.027) |  |  |  |
| Adjuvant treatment |  | 0.371 |  |  |
| Yes | Reference |  |  |  |
| No | 0.868 (0.636-1.184) |  |  |  |
